# Cannabidiol in Humans—The Quest for Therapeutic Targets

**DOI:** 10.3390/ph5050529

**Published:** 2012-05-21

**Authors:** Simon Zhornitsky, Stéphane Potvin

**Affiliations:** 1 Multiple Sclerosis Clinic, Foothills Medical Centre, Department of Clinical Neurosciences, Faculty of Medicine, University of Calgary, Calgary, Alberta T2N 1N4, Canada; 2 Fernand-Seguin Research Centre, Department of Psychiatry, Faculty of Medicine, Université de Montréal, Montreal, Quebec H1N 3V2, Canada

**Keywords:** cannabidiol, THC, cannabis, multiple sclerosis, pain, social anxiety disorder, epilepsy, insomnia, schizophrenia

## Abstract

Cannabidiol (CBD), a major phytocannabinoid constituent of cannabis, is attracting growing attention in medicine for its anxiolytic, antipsychotic, antiemetic and anti-inflammatory properties. However, up to this point, a comprehensive literature review of the effects of CBD in humans is lacking. The aim of the present systematic review is to examine the randomized and crossover studies that administered CBD to healthy controls and to clinical patients. A systematic search was performed in the electronic databases PubMed and EMBASE using the key word “cannabidiol”. Both monotherapy and combination studies (e.g., CBD + ∆9-THC) were included. A total of 34 studies were identified: 16 of these were experimental studies, conducted in healthy subjects, and 18 were conducted in clinical populations, including multiple sclerosis (six studies), schizophrenia and bipolar mania (four studies), social anxiety disorder (two studies), neuropathic and cancer pain (two studies), cancer anorexia (one study), Huntington’s disease (one study), insomnia (one study), and epilepsy (one study). Experimental studies indicate that a high-dose of inhaled/intravenous CBD is required to inhibit the effects of a lower dose of ∆9-THC. Moreover, some experimental and clinical studies suggest that oral/oromucosal CBD may prolong and/or intensify ∆9-THC-induced effects, whereas others suggest that it may inhibit ∆9-THC-induced effects. Finally, preliminary clinical trials suggest that high-dose oral CBD (150–600 mg/d) may exert a therapeutic effect for social anxiety disorder, insomnia and epilepsy, but also that it may cause mental sedation. Potential pharmacokinetic and pharmacodynamic explanations for these results are discussed.

## 1. Introduction

The cannabis plant has been used by humans for thousands of years in medicine for its sedative/hypnotic, antidepressant, analgesic, anticonvulsant, antiemetic, anti-inflammatory, anti-spasmodic and appetite-stimulating effects [1]. The plant is composed of a complex chemical mixture that includes phytocannabinoids, terpenoids, flavanoids, steroids and enzymes [2]. Phytocannabinoids—the most cannabis-specific of these constituents—bind to receptor sites normally activated by endogenous cannabinoids such as anadamide and 2-arachidonylglycerol (2-AG). It is widely believed the most psychoactive phytocannabinoid is delta-9-tetrahydrocannabinol (∆9-THC), which acts as a partial agonist at cannabinoid CB_1_ receptors—found primarily in the central nervous system (CNS), and CB_2_ receptors—found primarily on cells of the immune system [3,4]. However, apart from ∆9-THC, a number of other phytocannabinoids are present in significant quantities in cannabis (e.g., cannabidiol, cannabinol, cannabichromene), and they may be responsible for some of the plant’s many putative medicinal properties. In animal studies, cannabidiol (CBD) has been receiving growing attention for its antiemetic, anticonvulsant, antinflammatory, and antipsychotic properties [5,6,7,8]. This broad range of therapeutic effects may be a result of CBD’s complex pharmacological mechanisms [9]. Apart from ∆9-THC, CBD is the sole cannabinoid that has been thoroughly tested in humans in numerous controlled experimental studies as well as clinical trials for multiple sclerosis, neuropathic pain, schizophrenia, bipolar mania, social anxiety disorder, insomnia, Huntington’s disease and epilepsy. Surprisingly, however—up to this point—reviews and meta-analyses on the topic of CBD in humans have not considered a large number of experimental and clinical studies that administered CBD-alone and/or in combination with ∆9-THC, *versus* ∆9-THC-alone [10,11,12]. The inclusion of these studies is essential to understanding the therapeutic potential of CBD and its mediation by pharmacokinetic and pharmacodynamic factors.

The present review is aimed to comprehensively examine the effects of CBD in humans. We will begin with a brief overview of the pharmacokinetic and pharmacodynamic properties of CBD. Next, we will systematically examine the controlled experimental and clinical trials of CBD in order to elucidate its potential therapeutic role in human central nervous system (CNS) disorders.

## 2. Pharmacokinetics

CBD undergoes a significant first-pass effect leading to the formation of a number of metabolites, most notably, 7-hydroxy-CBD and CBD-7-oic acid [13,14]. The half-life of CBD in humans was found to be between 18–33 h following intravenous administration, 27–35 h following smoking, and 2–5 days following oral administration. Bioavailability of oral and smoked CBD in humans was found to be around 6% and 31%, respectively, providing further support for a substantial first-pass effect [13,15,16,17]. Oral administration of CBD (~700 mg) over six weeks to 14 Huntington’s disease patients resulted in a low, narrow plasma range of 5.9–11.2 ng/mL [15]. Oral cannabis extract (10 mg ∆9-THC; 10 mg CBD) produced markedly lower levels of CBD (range = 0–2.6 ng/mL) at 30–120 min after administration and absorption was increased with food [18,19].

Recent *in vitro* studies have shown that CBD is a potent inhibitor of multiple cytochrome P450 enzymes including CYP1A2, CYP2B6, CYP2C9, CYP2D6 and CYP3A4 [20,21,22,23]. Consequently, CBD may be expected to exhibit significant pharmacokinetic interaction with other pharmacological agents. In some studies, CBD has been shown to slightly augment levels of ∆9-THC (metabolized by CYP2C9, CYP2C19, and CYP3A4) by decreasing its conversion to 11-hydroxy-THC [19,24]. Moreover, animal studies found that CBD reduced the potency of some anticonvulsants and enhanced the potency of others; however, it is uncertain whether this effect resulted from a pharmacokinetic mechanism [25,26]. Pharmacokinetic interactions with other medications are probable, but studies are lacking.

## 3. Pharmacodynamics

CBD possesses affinity for CB_1_ and CB_2_ receptors in the micromolar range; however, despite this very low affinity, CBD seems to antagonize CB_1_/CB_2_ agonists with *K*_B_ values in the nanomolar range [9]. Some have suggested that the reason for these conflicting findings may be that CBD acts as a non-competitive inverse agonist, thereby blocking the ability of agonists to activate CB_1_/CB_2_ receptors [9]. Moreover, CBD has been found to antagonize the putative novel cannabinoid receptor GPR55, and the abnormal-CBD receptor at nanomolar concentrations [27,28]. In addition, there is evidence that CBD activates 5-HT_1A_ serotonergic and TRPV_1–2_ vanilloid receptors, antagonizes alpha-1 adrenergic and µ-opioid receptors, and inhibits synaptosomal uptake of noradrenaline, dopamine, serotonin and gaminobutyric acid and cellular uptake of anandamide at micromolar concentrations [29,30,31,32]. Studies also suggest that CBD may act on mitochondria Ca^2^ stores, block low-voltage-activated (T-type) Ca^2^ channels, and stimulate activity of the inhibitory glycine-receptor [33,34]. Finally, CBD has been shown to both stimulate and to inhibit activity of fatty amide hydrolase (FAAH; responsible for the degradation of anandamide) [35,36,37].

## 4. Methods

A systematic search was performed in the electronic databases PubMed and EMBASE using the key word “cannabidiol”. This search looked for human randomized and crossover studies published up to 1 April 2012. Both monotherapy and combination studies (e.g., CBD + ∆9-THC) were included. Studies that administered CBD in the form of cannabis cigarettes were included if the percentage of CBD was provided (studies which compared cannabis cigarettes with negligible amounts of CBD (<1%) were excluded). Pharmacokinetic studies and studies that only compared the combination of CBD/∆9-THC with placebo were excluded. Finally, studies that compared different routes of administration (e.g., oral *versus* oromucosal) were excluded.

## 5. Results

A total of 34 studies were identified. Sixteen of these were experimental studies, conducted in healthy subjects (Table 1) and 18 were conducted in clinical populations (Table 2). Of the clinical trials included patients with multiple sclerosis (six studies), schizophrenia and bipolar mania (four studies), social anxiety disorder (two studies), neuropathic and cancer pain (two studies), cancer anorexia (one study), Huntington’s disease (one study), insomnia (one study), and epilepsy (one study).

### 5.1. Experimental Studies in Healthy Controls

#### 5.1.1. Oral or Intravenous CBD-Alone

Six studies administered oral CBD-alone to healthy volunteers. An early study by Hollister [38] did not find any subjective or physiological effects with oral or intravenous CBD (100 mg PO and 30 mg IV) among 10 healthy volunteers. Additionally, a crossover study of oral CBD (200 mg) with, and without alcohol revealed no effect of the former on time production, finger tapping, cancellation test, and differential aptitude test [39]. There was also no difference in performance on these tests when CBD was added to alcohol, *versus* alcohol-alone; however, plasma alcohol levels in the CBD group were significantly lower compared to the alcohol-alone group. Another crossover study among 11 healthy volunteers revealed that plasma cortisol levels decreased during placebo treatment (in agreement with its normal circadian rhythm) and this decrease was attenuated by oral CBD (300 or 600 mg) [40]. Here, subjects reported CBD to have a sedative effect. A parallel-group study by the same authors compared the effects of diazepam, CBD (300 mg) and ipsapirone (a 5-HT1a agonist) among 40 individuals on anxiety before, during, and after a speech test [41]. Their results revealed that diazepam decreased anxiety before and after the speech test, whereas ipsapirone decreased it during, and CBD decreased it only after the speech test. More recently, a crossover study by Crippa *et al*. [42] showed that CBD (400 mg) decreased subjective anxiety and increased mental sedation among 10 healthy subjects, relative to placebo. Another crossover study found that treatment with 10 mg oral ∆9-THC increased levels of anxiety, intoxication, sedation, and psychotic symptoms among 15 participants, whereas CBD (600 mg) was inactive [43,44]. The authors also found that ∆9-THC increased the number of skin conductance response fluctuations during processing of intensely fearful faces, whereas CBD decreased it and there was a trend for reduced anxiety [45,46].

#### 5.1.2. Oral CBD/Ketamine

One crossover study examined the effects of oral CBD (600 mg) or placebo pretreatment on ketamine-induced psychiatric symptoms among 10 healthy volunteers [47]. Results revealed that significantly CBD increased ketamine-induced activation (as measured by the Brief Psychiatric Rating Scale, but failed to reduce ketamine-induced positive and negative symptoms, relative to placebo.

#### 5.1.3. Oral CBD/Nabilone

One crossover study examined the effects of oral CBD (200 mg) alone, and combined with nabilone (1 mg), relative to nabilone alone, in nine male subjects [48]. Here, CBD and nabilone caused mild sedation when administered alone. Moreover, CBD marginally reduced nabilone-induced intoxication and impairment in binocular depth perception—a model of impaired perception during psychotic states.

**Table 1:** Experimental studies.

Study	*N* (CBD)	Dosing	Outcome (≥ greater; ≤ less)
Hollister [38]	9 (5)	Fixed-dose; CBD 100 mg, PO; CBD 30 mg, IV	CBD = no subjective or physiological effects
Consroe *et al*. [39]	10 (10)	Fixed-dose; CBD 200 mg, PO	CBD = PBO (time production) CBD = PBO (finger tapping) CBD = PBO (cancellation test) CBD = PBO (differential aptitude test)
Zuardi *et al*. [40]	11 (11)	Fixed-dose; CBD 300 mg or 600 mg, PO	CBD > PBO (sedation) CBD < PBO (normal circadian decrease in cortisol level)
Zuardi *et al*. [41]	40 (10)	Fixed-dose; CBD 300 mg; DZP 10 mg; IPS 5 mg, PO	DZP < IPS < CBD < PBO (speech test-induced anxiety)
Crippa *et al*. [42]	10 (10)	Fixed-dose; CBD 400 mg, PO	CBD > PBO (mental sedation) CBD < PBO (anxiety)
Borgwardt *et al*. [43] Winton-Brown *et al*. [44] Fusar-Poli *et al*. [45,46]	15 (15)	Fixed-dose; CBD 600 mg; ∆9-THC 10 mg, PO	CBD < PBO < ∆9-THC (skin conductance response to fearful faces) CBD < PBO (anxiety *p* = 0.06) CBD = PBO (sedation, intoxication)
Hallak *et al*. [47]	10 (10)	Fixed dose; CBD 600 mg, PO; ketamine 0.25 mg/kg, IV	CBD > PBO (ketamine-induced activation [BPRS]) CBD = PBO (ketamine-induced positive and negative symptoms)
Leweke *et al*. [48]	9 (9)	Fixed-dose; CBD 200 mg; NAB 1 mg, PO	NAB > CBD + NAB > CBD (binocular depth perception deficit) CBD & NAB > PBO (sedation) NAB > CBD + NAB > CBD & PBO (intoxication)
Karniol *et al*. [49]	40 (5)	Fixed-dose; CBD 15 mg, 30 mg, 60 mg; ∆9-THC 30 mg, PO	CBD [15 mg] + ∆9-THC > ∆9-THC (pulse rate) CBD [30 & 60 mg] + ∆9-THC < ∆9-THC (pulse rate) ∆9-THC > CBD [all doses] + ∆9-THC (time production impairment)
Hollister and Gillespie [50]	15 (15)	Fixed-dose; CBD 40 mg; ∆9-THC 20 mg, PO	CBD + ∆9-THC > ∆9-THC (duration and intensity of intoxication) CBD + ∆9-THC > ∆9-THC (time to onset of intoxication) CBD + ∆9-THC = ∆9-THC (pulse rate)
Zuardi *et al*. [51]	8 (8)	Fixed-dose; CBD 1 mg/kg; ∆9-THC 0.5 mg/kg, PO	∆9-THC > CBD + ∆9-THC (anxiety, intoxication) CBD + ∆9-THC = ∆9-THC (pulse rate)
Juckel *et al*. [52] Roser *et al*. [53,54]	24 (24)	Fixed-dose; CBD 5.4 mg; ∆9-THC 10 mg, PO	CBD + ∆9-THC > ∆9-THC (MMN amplitude) CBD + ∆9-THC < PBO (right-hand tapping frequency) CBD + ∆9-THC = ∆9-THC (P300 amplitude)
Nicholson *et al*. [55]	8 (8)	Fixed-dose; CBD 15 mg; ∆9-THC 15 mg, OMC	CBD + ∆9-THC < ∆9-THC (impairment of immediate and delayed word recall) CBD + ∆9-THC = ∆9- THC (digit symbol substitution, choice reaction time, sustained attention, six-letter memory recall) CBD + ∆9-THC > ∆9-THC (awake time before sleep, sleepiness and fatigue upon awakening)
Dalton *et al*. [56]	15 (15)	Fixed-dose; CBD 150 µg/kg; ∆9-THC 25 µg/kg, INH	∆9-THC > CBD + ∆9-THC (intoxication) CBD + ∆9-THC & ∆9-THC > CBD (disturbance of stability of stance, motor performance, mental performance, manual coordination)
Ilan *et al*. [57]	23 (23)	Fixed-dose; CBD (1% *versus* 0.2%) ∆9-THC (3.6% *versus* 1.8%), INH	CBD + ∆9-THC = ∆9-THC (heart rate, intoxication) CBD [low] + ∆9-THC [high] > CBD [high] + ∆9-THC [high] (anxiety) CBD [high] + ∆9-THC [low] > CBD [low] + ∆9-THC [low] (anxiety)
Bhattacharyya *et al*. [58]	6 (6)	Fixed-dose; CBD 5 mg; ∆9-THC 1.25 mg, IV	∆9-THC > CBD + ∆9-THC (positive symptoms)

IPS = ipsapirone; DZP = diazepam; NAB = nabilone; OMC = oromucosal administration; PO = oral administration; CBD = cannabidiol; ∆9-THC = delta-9-tetrahydrocannabinol; IV = intravenous; BPRS = Brief Psychiatric Rating Scale; MMN = mismatch negativity; PBO = placebo.

**Table 2:** Clinical trials.

Study	*N* (CBD*)*	Subjects	Time	Dosing	Outcome(s) (≥ greater; ≤ less)
Consroe *et al*. [15]	15 (15)	Huntington’s	6 weeks	Flexible-dose; CBD 700 mg ^#^, PO	CBD = PBO (chorea severity)
Carlini and Cunha [ 59]	15 (15)	Insomnia	Acute	Fixed-dose; CBD 40 mg, 80 mg, 160 mg, NTZ 5 mg PO	CBD [160 mg] > PBO (sleep duration) CBD [all doses] < PBO (dream recall) CBD [all doses] = NTZ = PBO (sleep induction)
Cunha *et al*. [61]	15 (8)	Epilepsy	2–18 weeks	Flexible-dose; CBD 200–300 mg, PO	CBD < PBO (seizures)
Crippa *et al*. [62]	10 (10)	Social anxiety disorder	Acute	Fixed-dose; CBD 400 mg, PO	CBD < PBO (anxiety)
Bergamaschi *et al*. [63]	24 (12)	Social anxiety disorder	Acute	Fixed-dose; CBD 600 mg, PO	CBD < PBO (anxiety)
Leweke *et al*. [64]	42 (21)	Schizophrenia	4 weeks	Fixed-dose; CBD 600 mg; AMI 600 mg, PO	CBD = AMI (positive symptoms)
Zuardi *et al*. [65]	3 (3)	Schizophrenia	4 weeks	Fixed-dose; CBD—up to 1,280 mg, PO	CBD = PBO (positive and negative symptoms)
Zuardi *et al*. [66]	2 (2)	Bipolar I disorder	4 weeks	Fixed-dose; CBD—up to 1,280 mg, PO	CBD = PBO (mania)
Hallak *et al*. [67]	28 (9)	Schizophrenia	Acute	Fixed-dose; CBD 300 mg or 600 mg, PO	CBD [600 mg] > CBD [300 mg] & PBO (Stroop Color Word Test errors)
Killestein *et al*. [68,69]	16 (16)	Multiple sclerosis	4 weeks	Flexible-dose; ∆9-THC 5–10 mg; Cannabis extract 5–10 mg (20–30% CBD), PO	CBD + ∆9-THC > ∆9-THC > PBO (side-effects) CBD + ∆9-THC & ∆9-THC = PBO (spasticity) CBD + ∆9-THC > PBO (TNF-alpha)
Zajicek *et al*. [70] (CAMS)	630 (211)	Multiple sclerosis	15 weeks	Flexible-dose; CBD (to 12.5 mg/d); ∆9-THC (to 25 mg/d), PO	CBD + ∆9-THC & ∆9-THC = PBO (pain) CBD + ∆9-THC & ∆9-THC = PBO (spasticity) CBD + ∆9-THC & ∆9-THC = PBO (spasms) CBD + ∆9-THC & ∆9-THC = PBO (sleep quality)
Freeman *et al*. [71] (CAMS-LUTS)	255 (88)	Multiple sclerosis	13 weeks	Flexible-dose; CBD (to 12.5 mg/d); ∆9-THC (to 25 mg/d), PO	CBD + ∆9-THC & ∆9-THC < PBO (urinary incontinence)
Strasser *et al*. [72]	243 (95)	Cancer anorexia	6 weeks	Fixed-dose; CBD 2 mg; ∆9-THC 5 mg, PO	CBD + ∆9-THC & ∆9-THC = PBO (appetite, nausea, mood)
Johnson *et al*. [73]	177 (60)	Cancer pain	2 weeks	Flexible-dose; CBD 20–30 mg; ∆9-THC 22–32 mg, OMC	CBD + ∆9-THC < PBO (pain; NRS) ∆9-THC < PBO (pain; BPI-SF) CBD + ∆9-THC > PBO (nausea) CBD + ∆9-THC & ∆9-THC > PBO (cognitive deficits)
Brady *et al*. [74]	15 (15)	Multiple sclerosis	8 weeks	Flexible-dose; CBD & ∆9-THC 34 mg ^#^, OMC	∆9-THC < BAS (spasticity) ∆9-THC > BAS (sleep quality) CBD + ∆9-THC & ∆9-THC < BAS (pain) CBD + ∆9-THC & ∆9-THC < BAS (incontinence)
Wade *et al*. [75]	20 (20)	Multiple sclerosis (14/20) + neuropathic pain	2 weeks	Flexible-dose; CBD & ∆9-THC 45 mg ^#^, OMC	∆9-THC & CBD < PBO (pain; VAS) CBD + ∆9-THC & ∆9-THC & CBD = PBO (pain; NRS) CBD + ∆9-THC & ∆9-THC < PBO (spasms; VAS) ∆9-THC > PBO (appetite; VAS) CBD + ∆9-THC > PBO (sleep quality; VAS) ∆9-THC > PBO (memory impairment)
Notcutt *et al*. [76]	34 (34)	Multiple sclerosis (16/34) + neuropathic pain	5 weeks	Flexible-dose; CBD & ∆9-THC 2.5 mg per spray, OMC	CBD + ∆9-THC & ∆9-THC < CBD & PBO (pain) CBD + ∆9-THC & ∆9-THC > CBD > PBO (sleep quality)
Berman *et al*. [77]	48 (48)	Neuropathic pain	2 weeks	Flexible-dose; CBD & ∆9-THC 20 mg or 8–10 sprays per day ^#^, OMC	CBD + ∆9-THC & ∆9-THC < PBO (pain; BS-11) ∆9-THC < PBO (pain; SF-MPQ) CBD + ∆9-THC & ∆9-THC = PBO (pain disability) CBD + ∆9-THC & ∆9-THC > PBO (sleep quality)

CBD = cannabidiol; ∆9-THC = delta-9-tetrahyrdocannabinol; AMI = amisulpride; MS = multiple sclerosis; SAD = social anxiety disorder; OMC = oromucosal; PO = oral; PBO = placebo; IV = intravenous; INH = inhalation; ^#^ = mean dose; BPI-SF = Brief Pain Inventory Short Form; SF-MPQ = short-form McGill Pain Questionnaire; VAS = visual analogue scale; NRS = numerical rating scale; NTZ = nitrazepam; BAS = *versus* baseline value.

#### 5.1.4. Oral CBD/∆9-THC

Four studies administered oral CBD alone and/or together with oral ∆9-THC. A parallel-group study tested different doses of oral CBD (15, 30, 60 mg) alone, and combined with oral ∆9-THC (30 mg), relative to ∆9-THC-alone (30 mg) and placebo in 40 male subjects [49]. The authors found that, when given alone, CBD had little effect on pulse rate and psychological outcomes. Interestingly, there was a non-significant increase of 53% in pulse rate following the combination of CBD (15 mg) and ∆9-THC (30 mg); however, there was a significant decrease in pulse rate when the higher doses of CBD (30 and 60 mg) were combined with ∆9-THC. Furthermore, the 30 mg and 60 mg doses of CBD significantly attenuated ∆9-THC-induced increases in pulse rate as well as the number of “psychological reactions” (anxiety and panic), and all doses of CBD reversed ∆9-THC-induced impairment on a time estimation task. Moreover, a crossover study compared the addition of oral CBD (40 mg) to oral ∆9-THC (20 mg), relative to ∆9-THC-alone in 15 male subjects [50]. Results revealed that that CBD slightly increased time to onset, overall intensity, and duration of the subjective intoxication produced by oral ∆9-THC (20 mg), without affecting pulse rate. Another crossover study administered oral CBD (1 mg/kg) alone, and in combination with oral ∆9-THC (0.5 mg/kg) to eight male and female subjects [51]. The authors found that CBD had little effect on its own; however, it reduced ∆9-THC associated subjective intoxication and anxiety, without affecting pulse rate.

More recently, a crossover trial randomized 27 male and female subjects to treatment with oral CBD (5.4 mg) combined with oral ∆9-THC (10 mg), compared to ∆9-THC-alone and placebo [52,53,54]. First, the authors evidenced that the CBD/∆9-THC combination significantly increased auditory evoked mismatch negativity (MMN) amplitude, relative to placebo, whereas ∆9-THC-alone exerted no effect [52]. Second, they found that ∆9-THC treatment led to a reduction of P300 amplitude, but this effect was not reversed by CBD [53]. Third, they found that the CBD/∆9-THC combination, but not ∆9-THC-alone, reduced right-hand tapping frequencies *versus* placebo [54]. Finally, no significant differences were found for subjective intoxication or plasma levels of ∆9-THC or its metabolites in the subjects as a whole.

#### 5.1.5. Oromucosal CBD/∆9-THC

One crossover study investigated the effects of a combination of oromucosal CBD (15 mg) and ∆9-THC (15 mg), relative to ∆9-THC-alone (15 mg) on sleep and cognition in eight male and female subjects [55]. Measures were taken before sleep, during sleep and upon awakening. Results demonstrated that ∆9-THC-alone increased sleepiness 30 min after rising, and decreased latencies to early morning sleep, relative to placebo. The CBD/∆9-THC combination increased awake time before sleep, but also increased sleepiness and fatigue, compared to placebo. Lastly, no significant differences between the treatments were noticed on digit symbol substitution, choice reaction time, sustained attention, and six-letter memory recall. However, ∆9-THC-alone attenuated immediate word recall and delayed word recall, whereas the CBD/∆9-THC combination did not [55].

#### 5.1.6. Smoked or Intravenous CBD/∆9-THC

Three studies administered CBD/∆9-THC through smoking/intravenously. One early crossover study investigated the effects of smoked CBD (150 µg/kg) alone, and in conjunction with smoked ∆9-THC (25 µg/kg), relative to ∆9-THC-alone in 15 male subjects [56]. The authors found that CBD failed to exert any effects on its own and did not change ∆9-THC-induced increase in heart rate as well as impairment of stability of stance, motor performance, manual coordination and working memory. On the other hand, CBD decreased the “psychological high” associated with ∆9-THC (*p* < 0.05) [56]. Another crossover study administered cannabis cigarettes with various concentrations of ∆9-THC (3.6% *versus* 1.8%), CBD (1% *versus* 0.2%) and other minor cannabinoids to 23 male and female subjects [57]. They found CBD did not affect increases in heart rate and subjective intoxication produced by ∆9-THC. However, participants who received the lower dose of ∆9-THC tended to report more anxiety when paired with the higher dose CBD, relative to when paired with the lower dose CBD. By contrast, participants who received the higher ∆9-THC dose reported less anxiety when CBD content was high and more anxiety when CBD content was low [57]. Lastly, a crossover study administered CBD (5 mg) and ∆9-THC (1.25 mg) intravenously, relative to ∆9-THC-alone, in six male and female subjects [58]. The authors found that the addition of CBD blocked ∆9-THC-induced increases in Positive and Negative Syndrome Scale (PANSS) total scores.

### 5.2. Clinical Trials in Patient Populations

#### 5.2.1. Oral CBD-Alone

Nine clinical trials administered CBD-alone via the oral route. One early crossover trial comparing CBD (mean dose = 700 mg/d) with placebo among 15 Huntington’s disease patients did not find any significant differences in chorea severity, side-effects, clinical lab tests and other safety outcomes after 6-weeks of treatment [15]. On the other hand, a crossover trial comparing CBD (40, 80, and 160 mg) with placebo and nitrazepam (5 mg) among 15 insomniac volunteers revealed that duration of sleep significantly increased following administration of the high-dose CBD (160 mg); however, dream recall was reduced, relative to placebo [59,60]. The same authors subsequently conducted a 6-week, placebo-controlled, parallel-group study of CBD (200–300 mg/d), added to antiepileptic drugs, among 15 treatment-refractory epileptic patients [61]. Here, they found that four out of eight CBD-treated patients evidenced significant improvement in their condition, whereas only one patient improved in the placebo group.

More recently, a crossover study demonstrated that CBD (400 mg) decreased subjective anxiety among 10 treatment-naïve patients with social anxiety disorder, relative to placebo, and this was accompanied with significant changes in regional cerebral blood flow [62]. Similarly, a placebo-controlled, parallel-group study among 24 treatment-naive social anxiety disorder patients showed that CBD (600 mg) significantly reduced anxiety, cognitive impairment, and discomfort in speech performance, in response to a simulation public speaking test [63].

Preliminary data from a double-blind, randomized trial of 42 patients with acute schizophrenia revealed that CBD (600 mg) and amisulpride equally reduced psychotic symptoms after four weeks of treatment [64]. However, a placebo-controlled case-series did not find CBD to be effective among three treatment-resistant schizophrenia patients over four weeks [65]. Another placebo-controlled case-series by the same authors did not find a significant benefit of CBD for two bipolar mania patients after about four weeks of treatment [66]. Finally, a parallel-group among 28 schizophrenia patients that individuals who were treated with the low dose of CBD (300 mg) and placebo improved significantly more on the Stroop Color Word Test over two experimental sessions, relative to those treated with the high dose of CBD (600 mg) [67].

#### 5.2.2. Oral CBD/∆9-THC

Four clinical trials administered CBD/∆9-THC via the oral route. One crossover study treated 16 multiple sclerosis (MS) patients with flexible doses of cannabis extract (5–10 mg [20–30% CBD]), relative to ∆9-THC-alone (5–10 mg) over the course of four weeks. Results demonstrated that the CBD/∆9-THC combination resulted in significantly more adverse events (e.g., dizziness, somnolence, ataxia), compared to ∆9-THC-alone. No positive trends in efficacy (e.g., pain, tremor, spasticity, cognition) were noted for either of the treatments and they equally worsened participants’ global impressions, relative to placebo [68]. An immunological analysis revealed that patients who were treated with the CBD/THC combination evidenced a modest increase in TNF-alpha in LPS-stimulated whole blood and patients with high adverse event scores had an increase in plasma IL-12p40 [69]. Both of these immunomodulators have been linked with disease progression in MS. In addition, a parallel-group study treated 630 MS patients (Cannabinoids in Multiple Sclerosis [CAMS] study) with flexible doses of oral CBD (up to 12.5 mg/d) combined with ∆9-THC (up to 25 mg/d), *versus* ∆9-THC-alone and placebo [70]. The authors noted no evidence for a distinction between the treatments in efficacy (e.g., pain, tremor, spasticity, sleep) or adverse events, except for a tendency for CBD/∆9-THC to increase gastrointestinal side-effects, relative to ∆9-THC-alone. A sub-analysis showed that CBD/∆9-THC and ∆9-THC-alone significantly reduced urinary incontinence *versus* placebo [71]. On the other hand, a parallel-group study that treated 243 cancer-related anorexia patients with fixed doses of oral CBD (2 mg/d) and ∆9-THC (5 mg/d), relative to ∆9-THC-alone and placebo was terminated at interim analysis due to lack of difference between study arms [72].

#### 5.2.3. Oromucosal CBD/∆9-THC

Five trials administered CBD/∆9-THC via oromucosal sublingual drops. A parallel-group study treated 177 patients with cancer-related intractable pain with flexible doses of oromucosal CBD (20–30 mg/d) and ∆9-THC (22–32 mg/d), relative to ∆9-THC-alone and placebo [73]. Results showed that the combination of CBD and ∆9-THC was significantly better than placebo at decreasing pain on the neurological rating scale (NRS), whereas ∆9-THC-alone showed a non-significant reduction. By contrast, ∆9-THC-alone was more efficient than placebo at decreasing mean total pain on the Brief Pain Inventory–Short Form (BPI-SF; last 24 h) and no significant differences were found in EORTC Quality Of Life questionnaire (QLQ-C30) pain subscore or the amount of breakthrough opiate medication that was required. Additionally, CBD/∆9-THC increased nausea and vomiting on the QLQ-C30 subscore, but not on the NRS, relative to placebo. Finally, no significant differences were noted on the NRS memory or concentration subscores, or on the QLQ-C30 cognitive functioning subscore [73]. In addition, a crossover trial that treated 15 MS patients with a 1:1 ratio of CBD/∆9-THC (mean = 34 mg/d), relative to ∆9-THC -alone revealed that patients preferred ∆9-THC-alone because they found it more effective for controlling urinary symptoms and needed less of it to achieve a therapeutic effect [74]. Analysis of secondary outcome measures revealed that ∆9-THC-alone was significantly better than CBD/∆9-THC for spasticity and sleep but both treatments equally improved VAS pain scores [74]. Another crossover trial treated 24 patients with MS and neuropathic pain with CBD/∆9-THC (1:1 ratio; 2.5 mg per spray), relative to CBD-alone, ∆9-THC-alone and placebo [75]. Analysis of data showed that CBD-alone and ∆9-THC-alone significantly improved VAS pain scores *versus* placebo; however, none of the treatments improved pain as measured by the NRS. Moreover, both CBD/∆9-THC and ∆9-THC-alone improved spasm, but only the latter improved spasticity and appetite, whereas only the former improved sleep quality. Lastly, administration of ∆9-THC-alone produced the greatest subjective intoxication and reduction in Short Orientation-Memory-Concentration test score, relative to placebo [75].

Furthermore, a crossover trial treated 34 patients with MS and neuropathic pain CBD/∆9-THC (1:1 ratio; 2.5 mg per spray), compared to CBD-alone, ∆9-THC-alone and placebo [76]. Results revealed that CBD/∆9-THC and ∆9-THC-alone were equally beneficial for pain and all three treatments (including CBD-alone, but less so) improved sleep quality, relative to placebo. Of the 28 patients that obtained benefit, 14 found CBD/∆9-THC and ∆9-THC equally satisfactory, 11 preferred CBD/∆9-THC, two preferred ∆9-THC-alone, and one found ∆9-THC-alone and CBD-alone equally satisfactory [76]. An additional crossover trial treated 48 neuropathic pain patients (brachial plexus avulsion) with CBD/∆9-THC (1:1 ratio; 2.5 mg per spray), compared to ∆9-THC-alone and placebo [77]. The authors found that the combination of ∆9-THC-alone (but not CBD/∆9-THC) significantly decreased pain on the short form McGill Questionnaire, relative to placebo. However, both treatments significantly decreased pain ratings on an 11-point Box Scale and neither treatment significantly decreased pain on the Pain Disability Index. Finally, both treatments equally improved sleep quality.

## 6. Discussion

Experimental studies suggest that high-dose CBD may decrease anxiety and increase mental sedation in healthy individuals. Clinical trials suggest the high-dose CBD may be useful for the treatment of social anxiety disorder, and possibly, insomnia and epilepsy. The anxiolytic effect associated with CBD may be the result of its 5-HT_1A_ agonism, which has been evidenced in a number of behavioral studies [78,79,80,81]. Paradoxically, some animal studies have found that the dose-response of CBD follows an inverted U shape, leading to an anxiogenic effect through its agonism of TRPV_1–2_ receptors (which are believed to be responsible for detection and regulation of body temperature, and thermal nociception) [82]. Alternatively, it is possible that the anxiolytic properties of CBD are mediated by its action at CB_1_ receptors, because CB_1_ antagonists were found to attenuate amphetamine and/or nicotine-induced anxiety in mice [83]. Indeed, there is evidence that both CBD and AM404 (an anandamide transporter/FAAH inhibitor and TRPV_1_ agonist) facilitated extinction of contextual fear memory in rats and this was reversed by the CB_1_-receptor antagonist SR141716A, but not by the TRPV_1_-selective antagonist, capsazepine [84]. It is equally possible that the anxiolytic effect of CBD is explained by inhibition of FAAH. Animal studies have demonstrated that FAAH inhibitors possess anxiolytic properties in a number of paradigms including marble burying, light/dark box, elevated zero maze, and isolation-induced ultrasonic emission test [85,86,87]. However, CBD has been shown to both inhibit and stimulate activity FAAH [35,36,37]. Consequently, it is yet unclear whether there is a role for inhibition of endocannabinoid catabolic enzymes in the anxiolytic effects of CBD.

A possible analgesic effect of CBD-alone, and CBD added to ∆9-THC was observed in two studies among mixed neurogenic (MS and neuropathic pain) and cancer pain patients [73,75]. However, both studies administered low doses of CBD (2.5 mg CBD and ∆9-THC per spray), used more than one scale to measure pain outcomes and their results were not consistent across scales. Also confounding interpretation is the fact that some of the clinical trials allowed the use of “rescue medication”, which may have led overestimation of the effects of CBD-alone [75,76]. In support, other studies did not find an analgesic effect of CBD-alone, or in combination with ∆9-THC [70,74,77]. In animals, there is evidence that ∆9-THC and cannabinol (a weak partial CB_1_ agonist) suppressed the abdominal constriction response to formic acid in mice, whereas CBD was inactive at doses of up to 200 mg/kg [88]. In that study, the analgesic effects of ∆9-THC and cannabinol were additive and CBD antagonised these effects in a dose-dependent manner. Likewise, ∆9-THC, cannabinol and cannabis extract produced an analgesic effect in the hot-plate test in mice; however, CBD was without effect at doses of up to 30 mg/kg [89]. By contrast, CBD (5 mg/kg IP or 25 mg/kg PO) was shown to block disease progression in murine collagen-induced arthritis—an animal model of rheumatoid arthritis—and this was accompanied by reductions in type-II collagen-specific proliferation, interferon—gamma production, and release of tumour necrosis factor-alpha by synovial cells [90]. The same group later demonstrated that the synthetic CBD derivative—HU-320—exerted more potent effects in the same direction [91]. More recently, there is evidence that cannabidiol derivative, O-1602, reduced movement-evoked firing of nociceptive C fibres in a rat model of acute inflammatory joint pain [92]. Interestingly, this effect was blocked by the GPR55-receptor antagonist, O-1918, but not by the CB_1_ and CB_2_ antagonists, AM281 and AM630, respectively. As a whole, these data indicate that CBD and its analogues may be beneficial for pain resulting from inflammation, however, human studies on this topic are lacking.

The strength of the antiepileptic effects of CBD may be difficult to judge clinically because of its potent antagonism of multiple CYP isoenzymes, potentially reducing plasma levels of concomitant anticonvulsants. Preclinical data has indicated that CBD displays antiepileptiform and antiseizure properties *in vitro* and *in vivo* CBD may possess antiepileptic properties via different mechanisms. For instance, there is evidence that CBD can block low-voltage-activated (T-type) Ca^2^ channels, and increase the activity of inhibitory glycine receptors [33,34]. More recently, Jones *et al*. [5] used extracellular multi-electrode array recordings to show that CBD decreased epileptiform activity in the Mg^2^-free and 4-aminopyridine *in vitro* models of hippocampal epilepsy in the mammalian hippocampus—a key epileptogenic brain region. Additionally, the authors examined the effects of CBD (1, 10, and 100 mg/kg) *in vivo* using the pentylenetetrazole model of generalized seizures. Their results revealed that the incidence of severe seizures and mortality was significantly attenuated in rats treated with the high dose of CBD (100 mg/kg), relative to vehicle-treated rats. The antiepileptic effects associated with CBD were suggested to be due a potentially CB_1_ independent mechanism because CBD acted with only low affinity at CB_1_ receptor and displayed no agonist activity in [^35^S]guanosine 5'-*O*-(3-thio)-triphosphate assays in cortical membranes. In support of this interpretation, there is evidence that the anticonvulsant properties of CBD in the maximal electroshock model were not affected by the CB_1_-recepetor antagonist, SR141716A, whereas those of ∆9-THC and the CB_1_-receptor agonist, WIN55,212-2, were blocked [93].

Despite its putative benefits for social anxiety disorder, insomnia and epilepsy studies suggest that high-dose CBD (400–700 mg) may increase mental sedation in normal individuals and aggravate cognitive deficits in schizophrenia—without altering physical sedation [42,62,67]. While the mechanism that is responsible for these effects is not clear, the fact that they exist is not surprising because most anxiolytics/sedatives/anticonvulsants produce their therapeutic action by decreasing CNS activation, and consequently, alertness. Some research does suggest, however, that CBD may improve cognition when used in combination with ∆9-THC. For instance, there is evidence that mixed neurogenic patients given oromucosal CBD/∆9-THC performed as well as patients given placebo on the Short Orientation-Memory-Concentration test, whereas patients given ∆9-THC-alone performed significantly worse than placebo-treated patients [75]. Similarly, there is evidence that healthy subjects given oromucosal CBD/∆9-THC (15 and 15 mg) performed equally well as placebo-treated individuals on tests of delayed and immediate word recall, whereas subjects treated with ∆9-THC-alone performed significantly worse than placebo-treated subjects on those tasks and they exhibited less wakefulness [55]. However, the treatments were not significantly different from placebo on digit symbol substitution, choice reaction, sustained attention, six-letter memory recall, digit memory recall. Moreover, Karniol *et al.* [49] showedthat oral CBD (15, 30, and 60 mg) inhibited the time production impairment associated with ∆9-THC (30 mg) [49]. On the other hand, Dalton *et al*. [56] found that a high dose of smoked CBD (150 µg/kg) failed to block perturbations of stability of stance, motor performance, mental performance induced by a much lower dose of smoked Δ9-THC (25 µg/kg).

Current evidence is equivocal regarding a potential antipsychotic effect of CBD. For example, Zuardi *et al*. [65,66] did not find CBD monotherapy (up to 1,280 mg) to be effective relative to placebo in a case-series of bipolar mania and treatment-resistant schizophrenia patients. Likewise, the CB_1_ antagonist, SR141716, was ineffective for the treatment of positive or negative symptoms in schizophrenia [94]. In another study, oral CBD (600 mg) enhanced the psychomotor activating effects of intravenous ketamine, without significantly altering ketamine-induced psychiatric symptoms [47]. On the other hand, preliminary data from a four-week, randomized-controlled trial of CBD (600 mg) *versus* amisulpride (600 mg) for schizophrenia did not reveal any significant differences between the groups—suggesting that the former exerted an antipsychotic effect [64].

Intriguingly, some studies show that CBD can potentiate and some that that it can attenuate the psychotomimetic effects associated with Δ9-THC, depending on the measure, route of administration, and dose-ratio between the cannabinoids. For example, Karniol *et al*. [49] found that a low dose of CBD potentiated Δ9-THC-induced increases in pulse rate, whereas an equal or higher dose of CBD attenuated these increases. A dose-dependent interaction was also evidenced in the study by Ilan *et al*. [57] wherein high doses of CBD potentiated anxiety induced by low doses of Δ9-THC, but they reduced anxiety induced by high doses of Δ9-THC. In addition, Hollister and Gallespie [50] found that oral CBD (40 mg) caused a slight delay and prolongation/intensification of the psychotomimetic effects of Δ9-THC (20 mg). By contrast, Dalton *et al*. [56] showed that a high dose of smoked CBD (150 µg/kg) minimally (*p* < 0.05) inhibited the psychotomimetic effects associated with smoked Δ9-THC (25 µg/kg). However, there is also evidence that large doses of intravenous CBD (5 mg) completely blocked elevations in PANSS positive symptoms induced by intravenous Δ9-THC (1.25 mg) [58]. Another group found significantly greater MMN amplitude values at central electrodes following treatment with combined CBD (5.4 mg) and Δ9-THC (10 mg; but not Δ9-THC-alone), indicating that the former may have exerted an antipsychotic effect [52]. Finally, a study using nabilone (1 mg) showed that the molecule significantly impaired binocular depth inversion (an illusion of visual perception that provides a model of impaired perception during psychotic states) and this effect was partially reversed by CBD (200 mg) [48].

Overall, the human data regarding CBD’s potential to reverse the cognitive perturbations and psychotomimetic symptoms induced by Δ9-THC are difficult to interpret due to the possibility of a pharmacokinetic interaction between CBD and Δ9-THC (or other molecules) following oral/oromucosal administration. A review of 1970s studies found that the ratio of CBD/Δ9-THC was 8.1 when the CBD displayed antagonistic effects and 1.8 when it enhanced the effects of Δ9-THC [95,96]. Moreover, there is evidence that combination of CBD (1–10 mg/kg IP over 21 days) with equivalent doses of ∆9-THC increased blood and brain levels of the latter, decreased levels of 11-OH-THC and THC-COOH, and augmented the anxiogenic and locomotor suppressant effects and social interaction deficits seen with ∆9-THC [97]. Interestingly, CBD did not change the THC-induced decrease in CB_1_ receptor binding and none of the treatments altered 5-HT_1A_ binding, suggesting that pharmacokinetic factors may have played a role.

The presence of a pharmacokinetic interaction between CBD and Δ9-THC is supported by results of two early phase studies of nabiximols—an oromucosal spray that contains an equivalent dose of the cannabinoids [98,99]. For instance, the addition of CBD (20 mg) to ∆9-THC (20 mg) significantly increased the area under-the-curve (AUC) of 11-hydroxy-THC [98]. The same group also demonstrated that concomitant CBD (10 mg) and ∆9-THC (10 mg) lead to a significantly later *T*_max_ for ∆9-THC, relative to treatment with ∆9-THC-alone [99]. These pharmacokinetic data roughly correspond to mean intoxication ratings across time, indicating that CBD slightly delayed and prolonged the subjective effects associated with ∆9-THC. By contrast, Nadulski *et al*. [18,19] found that oral CBD (5.4 mg), combined with ∆9-THC (10 mg) (non-significantly) increased the AUC and maximum concentration of ∆9-THC by approximately 20%, suggesting that CBD inhibited the conversion of ∆9-THC into 11-hydroxy-THC. Nevertheless, the pharmacokinetic impact of CBD was small compared to other factors such as gender and body mass index.

On the other hand, it remains true that a few human studies showed that oral/oromucosal CBD attenuated the psychoactive and therapeutic effects associated with ∆9-THC at low doses and dose-ratios between the cannabinoids [49,55,74,75]. Indeed, Karniol *et al*. [49] found that oral CBD (15 mg) was sufficient to reverse ∆9-THC-induced (30 mg) impairment on a time estimation task. In a similar fashion, there is evidence that oromucosal CBD (15 mg) attenuated verbal memory deficits induced by ∆9-THC (15 mg) [55]. Clinical studies using equal ratios of CBD to ∆9-THC show that the former may alter both the benefits and side-effects associated with the latter. For instance, CBD attenuated the antiemetic effects of ∆9-THC in cancer patients [73]. Moreover, CBD attenuated the antispastic, memory-impairing, and intoxicating effects associated with ∆9-THC and increased the prevalence of adverse events among MS patients (e.g., dizziness, ataxia, gastrointestinal) [68,71,73,74,75]. Nonetheless, clinical trials did not consistently show significant benefit/drawbacks of combining CBD with ∆9-THC. Some of the variability in results may be attributed to the fact that studies contained small sample sizes and measured multiple variables, leading to the possibility of a Type-I error(s). Likewise, some variability may explained by the fact that a number of clinical trials treated patients with CBD/Δ9-THC during a “run-in” period, which may have biased results towards CBD/Δ9-THC because individuals who did not respond or could not tolerate the medication would have withdrawn early [74,75,76]. Alternatively, CBD may exhibit a flat dose-response curve, whereby all doses are able to partially reverse the effects of Δ9-THC because of its non-competitive antagonist action at CB_1_ receptors [9]. A final explanation for the disparate results is that oral CBD has the ability to attenuate/potentiate some and (but not other) effects associated with Δ9-THC due to activity at receptors other than CB_1_, even at low doses and small ratios of CBD/Δ9-THC. Such an effect may be one reason why some studies found contradictory results using similar dose-ratios between the cannabinoids [51,55].

## 7. Conclusions

Experimental studies indicate that a high-dose of inhaled/intravenous CBD is required to inhibit the effects of a lower dose of ∆9-THC. Some experimental and clinical studies also suggest that oral/oromucosal CBD may prolong and/or intensify ∆9-THC-induced effects, whereas others suggest that it may inhibit ∆9-THC-induced effects. A balance between pharmacokinetic and pharmacodynamic factors may be responsible for the disparate findings, depending on the measure, route of administration and dose-ratio between the cannabinoids. Moreover, preliminary clinical trials suggest that high-dose oral CBD (150–600 mg/d) may exert a therapeutic effect for social anxiety disorder, insomnia and epilepsy, but also that it may cause mental sedation. On the other hand, trials did not consistently observe any benefits/drawbacks of adding low-dose CBD to ∆9-THC for clinical conditions such as MS, neuropathic and cancer pain, and cancer-associated anorexia. Likewise, studies did not consistently observe benefits of CBD monotherapy in bipolar mania or schizophrenia patients.

Future studies should investigate clinical applications of high-dose oral CBD for disorders such as anxiety, neuropathic pain, inflammatory pain, multiple sclerosis, insomnia and epilepsy. Future trials should also administer CBD to clinical patients for prolonged periods of time in order to simulate the “real world” setting. If CBD is not found to be beneficial in these trials, new more selective and more bioavailable molecules need to be developed in order to harness the full therapeutic potential of cannabinoid molecules. Currently, the most promising candidates are inhibitors of endocannabinoid catabolic enzymes (e.g., anandamide, FAAH) for the treatment of anxiety and depressive disorders [100].

## References

[1] Zuardi A.W. (2006). History of cannabis as a medicine: A review. Rev. Bras. Psiquiatr..

[2] Elsohly M.A., Slade D. (2005). Chemical constituents of marijuana: The complex mixture of natural cannabinoids. Life Sci..

[3] Ameri A. (1999). The effects of cannabinoids on the brain. Prog. Neurobiol..

[4] Desfosses J., Stip E., Bentaleb L.A., Potvin S. (2010). Endocannabinoids and schizophrenia. Pharmaceuticals.

[5] Jones N.A., Hill A.J., Smith I., Bevan S.A., Williams C.M., Whalley B.J., Stephens G.J. (2010). Cannabidiol displays antiepileptiform and antiseizure properties *in vitro* and *in vivo*. J. Pharmacol. Exp. Ther..

[6] Roser P., Vollenweider F.X., Kawohl W. (2010). Potential antipsychotic properties of central cannabinoid (CB1) receptor antagonists. World J. Biol. Psychiatry.

[7] Booz G.W. (2011). Cannabidiol as an emergent therapeutic strategy for lessening the impact of inflammation on oxidative stress. Free Radic. Biol. Med..

[8] Rock E.M., Goodwin J.M., Limebeer C.L., Breuer A., Pertwee R.G., Mechoulam R., Parker L.A. (2011). Interaction between non-psychotropic cannabinoids in marihuana: Effect of cannabigerol (CBG) on the anti-nausea or anti-emetic effects of cannabidiol (CBD) in rats and shrews. Psychopharmacology (Berl.).

[9] Pertwee R.G. (2008). The diverse CB1 and CB2 receptor pharmacology of three plant cannabinoids: delta9-Tetrahydrocannabinol, cannabidiol and delta9-tetrahydrocannabivarin. Br. J. Pharmacol..

[10] Iskedjian M., Bereza B., Gordon A., Piwko C., Einarson T.R. (2007). Meta-analysis of cannabis based treatments for neuropathic and multiple sclerosis-related pain. Curr. Med. Res. Opin..

[11] Zuardi A.W. (2008). Cannabidiol: From an inactive cannabinoid to a drug with wide spectrum of action. Rev. Bras. Psiquiatr..

[12] Iuvone T., Esposito G., de Filippis D., Scuderi C., Steardo L. (2009). Cannabidiol: A promising drug for neurodegenerative disorders?. CNS Neurosci. Ther..

[13] Agurell S., Halldin M., Lindgren J.E., Ohlsson A., Widman M., Gillespie H., Hollister L. (1986). Pharmacokinetics and metabolism of delta 1-tetrahydrocannabinol and other cannabinoids with emphasis on man. Pharmacol. Rev..

[14] Harvey D.J., Mechoulam R. (1990). Metabolites of cannabidiol identified in human urine. Xenobiotica.

[15] Consroe P., Laguna J., Allender J., Snider S., Stern L., Sandyk R., Kennedy K., Schram K. (1991). Controlled clinical trial of cannabidiol in Huntington’s disease. Pharmacol. Biochem. Behav..

[16] Ohlsson A., Lindgren J.E., Andersson S., Agurell S., Gillespie H., Hollister L.E. (1986). Single-dose kinetics of deuterium-labelled cannabidiol in man after smoking and intravenous administration. Biomed. Environ. Mass Spectrom..

[17] Guy G.W., Whittle B.A., Robson P. (2004). The Medicinal Uses of Cannabis and Cannabinoids.

[18] Nadulski T., Pragst F., Weinberg G., Roser P., Schnelle M., Fronk E.M., Stadelmann A.M. (2005). Randomized, double-blind, placebo-controlled study about the effects of cannabidiol (CBD) on the pharmacokinetics of delta-9-tetrahydrocannabinol (THC) after oral application of THC verses standardized cannabis extract. Ther. Drug Monit..

[19] Nadulski T., Sporkert F., Schnelle M., Stadelmann A.M., Roser P., Schefter T., Pragst F. (2005). Simultaneous and sensitive analysis of THC, 11-OH-THC, THC-COOH, CBD, and CBN by GC-MS in plasma after oral application of small doses of THC and cannabis extract. J. Anal. Toxicol..

[20] Yamaori S., Kushihara M., Yamamoto I., Watanabe K. (2010). Characterization of major phytocannabinoids, cannabidiol and cannabinol, as isoform-selective and potent inhibitors of human CYP1 enzymes. Biochem. Pharmacol..

[21] Yamaori S., Ebisawa J., Okushima Y., Yamamoto I., Watanabe K. (2011). Potent inhibition of human cytochrome P450 3A isoforms by cannabidiol: Role of phenolic hydroxyl groups in the resorcinol moiety. Life Sci..

[22] Yamaori S., Okamoto Y., Yamamoto I., Watanabe K. (2011). Cannabidiol, a major phytocannabinoid, as a potent atypical inhibitor for CYP2D6. Drug Metab. Dispos..

[23] Yamaori S., Koeda K., Kushihara M., Hada Y., Yamamoto I., Watanabe K. (2011). Comparison in the *in vitro* inhibitory effects of major phytocannabinoids and polycyclic aromatic hydrocarbons contained in marijuana smoke on cytochrome P450 2C9 activity. Drug Metab. Pharmacokinet..

[24] Hunt C.A., Jones R.T., Herning R.I., Bachman J. (1981). Evidence that cannabidiol does not significantly alter the pharmacokinetics of tetrahydrocannabinol in man. J. Pharmacokinet. Biopharm..

[25] Turkanis S.A., Cely W., Olsen D.M., Karler R. (1974). Anticonvulsant properties of cannabidiol. Res. Commun. Chem. Pathol. Pharmacol..

[26] Consroe P., Wolkin A. (1977). Cannabidiol-antiepileptic drug comparisons and interactions in experimentally induced seizures in rats. J. Pharmacol. Exp. Ther..

[27] Ryberg E., Larsson N., Sjögren S., Hjorth S., Hermansson N.O., Leonova J., Elebring T., Nilsson K., Drmota T., Greasley P.J. (2007). The orphan receptor GPR55 is a novel cannabinoid receptor. Br. J. Pharmacol..

[28] Járai Z., Wagner J.A., Varga K., Lake K.D., Compton D.R., Martin B.R., Zimmer A.M., Bonner T.I., Buckley N.E., Mezey E. (1999). Cannabinoid-induced mesenteric vasodilation through an endothelial site distinct from CB1 or CB2 receptors. Proc. Natl. Acad. Sci. USA.

[29] Russo E.B., Burnett A., Hall B., Parker K.K. (2005). Agonistic properties of cannabidiol at 5-HT1a receptors. Neurochem. Res..

[30] Pertwee R.G., Ross R.A., Craib S.J., Thomas A. (2002). (−)-Cannabidiol antagonizes cannabinoid receptor agonists and noradrenaline in the mouse vas deferens. Eur. J. Pharmacol..

[31] Bisogno T., Hanus L., de Petrocellis L., Tchilibon S., Ponde D.E., Brandi I., Moriello A.S., Davis J.B., Mechoulam R., di Marzo V. (2001). Molecular targets for cannabidiol and its synthetic analogues: Effect on vanilloid VR1 receptors and on the cellular uptake and enzymatic hydrolysis of anandamide. Br. J. Pharmacol..

[32] de Petrocellis L., Ligresti A., Moriello A.S., Allarà M., Bisogno T., Petrosino S., Stott C.G., di Marzo V. (2011). Effects of cannabinoids and cannabinoid-enriched Cannabis extracts on TRP channels and endocannabinoid metabolic enzymes. Br. J. Pharmacol..

[33] Ross H.R., Napier I., Connor M. (2008). Inhibition of recombinant human T-type calcium channels by Delta9-tetrahydrocannabinol and cannabidiol. J. Biol. Chem..

[34] Ahrens J., Demir R., Leuwer M., de la Roche J., Krampfl K., Foadi N., Karst M., Haeseler G. (2009). The nonpsychotropic cannabinoid cannabidiol modulates and directly activates alpha-1 and alpha-1-beta glycine receptor function. Pharmacology.

[35] Watanabe K., Ogi H., Nakamura S., Kayano Y., Matsunaga T., Yoshimura H., Yamamoto I. (1998). Distribution and characterization of anandamide amidohydrolase in mouse brain and liver. Life Sci..

[36] Massi P., Valenti M., Vaccani A., Gasperi V., Perletti G., Marras E., Fezza F., Maccarrone M., Parolaro D. (2008). 5-Lipoxygenase and anandamide hydrolase (FAAH) mediate the antitumor activity of cannabidiol, a non-psychoactive cannabinoid. J. Neurochem..

[37] de Petrocellis L., di Marzo V. (2010). Non-CB1, non-CB2 receptors for endocannabinoids, plant cannabinoids, and synthetic cannabimimetics: Focus on G-protein-coupled receptors and transient receptor potential channels. J. Neuroimmune Pharmacol..

[38] Hollister L.E. (1973). Cannabidiol and cannabinol in man. Experientia.

[39] Consroe P., Carlini E.A., Zwicker A.P., Lacerda L.A. (1979). Interaction of cannabidiol and alcohol in humans. Psychopharmacology (Berl.).

[40] Zuardi A.W., Guimarães F.S., Moreira A.C. (1993). Effect of cannabidiol on plasma prolactin, growth hormone and cortisol in human volunteers. Braz. J. Med. Biol. Res..

[41] Zuardi A.W., Cosme R.A., Graeff F.G., Guimarães F.S. (1993). Effects of ipsapirone and cannabidiol on human experimental anxiety. J. Psychopharmacol..

[42] Crippa J.A., Zuardi A.W., Garrido G.E., Wichert-Ana L., Guarnieri R., Ferrari L., Azevedo-Marques P.M., Hallak J.E., McGuire P.K., Filho Busatto G. (2004). Effects of cannabidiol (CBD) on regional cerebral blood flow. Neuropsychopharmacology.

[43] Borgwardt S.J., Allen P., Bhattacharyya S., Fusar-Poli P., Crippa J.A., Seal M.L., Fraccaro V., Atakan Z., Martin-Santos R., O’Carroll C. (2008). Neural basis of delta-9-tetrahydrocannabinol and cannabidiol: Effects during response inhibition. Biol. Psychiatry.

[44] Winton-Brown T.T., Allen P., Bhattacharyya S., Borgwardt S.J., Fusar-Poli P., Crippa J.A., Seal M.L., Martin-Santos R., Ffytche D., Zuardi A.W. (2011). Modulation of auditory and visual processing by delta-9-tetrahydrocannabinol and cannabidiol: An FMRI study. Neuropsychopharmacology..

[45] Fusar-Poli P., Crippa J.A., Bhattacharyya S., Borgwardt S.J., Allen P., Martin-Santos R., Seal M., Surguladze S.A., O’Carrol C., Atakan Z. (2009). Distinct effects of {delta}9-tetrahydrocannabinol and cannabidiol on neural activation during emotional processing. Arch. Gen. Psychiatry.

[46] Fusar-Poli P., Allen P., Bhattacharyya S., Crippa J.A., Mechelli A., Borgwardt S., Martin-Santos R., Seal M.L., O’Carrol C., Atakan Z. (2010). Modulation of effective connectivity during emotional processing by delta-9-tetrahydrocannabinol and cannabidiol. Int. J. Neuropsychopharmacol..

[47] Hallak J.E., Dursun S.M., Bosi D.C., de Macedo L.R., Machado-de-Sousa J.P., Abrão J., Crippa J.A., McGuire P., Krystal J.H., Baker G.B. (2011). The interplay of cannabinoid and NMDA glutamate receptor systems in humans: Preliminary evidence of interactive effects of cannabidiol and ketamine in healthy human subjects. Prog. Neuropsychopharmacol. Biol. Psychiatry.

[48] Leweke F.M., Schneider U., Radwan M., Schmidt E., Emrich H.M. (2000). Different effects of nabilone and cannabidiol on binocular depth inversion in man. Pharmacol. Biochem. Behav..

[49] Karniol I.G., Shirakawa I., Kasinski N., Pfeferman A., Carlini E.A. (1974). Cannabidiol interferes with the effects of delta 9-tetrahydrocannabinol in man. Eur. J. Pharmacol..

[50] Hollister L.E., Gillespie H. (1975). Interactions in man of delta-9-tetrahydrocannabinol. II. Cannabinol and cannabidiol. Clin. Pharmacol. Ther..

[51] Zuardi A.W., Shirakawa I., Finkelfarb E., Karniol I.G. (1982). Action of cannabidiol on the anxiety and other effects produced by delta 9-THC in normal subjects. Psychopharmacology (Berl.).

[52] Juckel G., Roser P., Nadulski T., Stadelmann A.M., Gallinat J. (2007). Acute effects of Delta9-tetrahydrocannabinol and standardized cannabis extract on the auditory evoked mismatch negativity. Schizophr. Res..

[53] Roser P., Juckel G., Rentzsch J., Nadulski T., Gallinat J., Stadelmann A.M. (2008). Effects of acute oral delta-9-tetrahydrocannabinol and standardized cannabis extract on the auditory P300 event-related potential in healthy volunteers. Eur. Neuropsychopharmacol..

[54] Roser P., Gallinat J., Weinberg G., Juckel G., Gorynia I., Stadelmann A.M. (2009). Psychomotor performance in relation to acute oral administration of delta-9-tetrahydrocannabinol and standardized cannabis extract in healthy human subjects. Eur. Arch. Psychiatry Clin. Neurosci..

[55] Nicholson A.N., Turner C., Stone B.M., Robson P.J. (2004). Effect of delta-9-tetrahydrocannabinol and cannabidiol on nocturnal sleep and early-morning behavior in young adults. J. Clin. Psychopharmacol..

[56] Dalton W.S., Martz R., Lemberger L., Rodda B.E., Forney R.B. (1976). Influence of cannabidiol on delta-9-tetrahydrocannabinol effects. Clin. Pharmacol. Ther..

[57] Ilan A.B., Gevins A., Coleman M., ElSohly M.A., de Wit H. (2005). Neurophysiological and subjective profile of marijuana with varying concentrations of cannabinoids. Behav. Pharmacol..

[58] Bhattacharyya S., Morrison P.D., Fusar-Poli P., Martin-Santos R., Borgwardt S., Winton-Brown T., Nosarti C., O’Carroll C.M., Seal M., Allen P. (2010). Opposite effects of delta-9-tetrahydrocannabinol and cannabidiol on human brain function and psychopathology. Neuropsychopharmacology.

[59] Carlini E.A., Masur J., Magalhaes C.C.P.B. (1979). Possvel efeito hipnotico do cannabidiol no ser humano. Estudo preliminar. Cienc. Cult..

[60] Carlini E.A., Cunha J.M. (1981). Hypnotic and antiepileptic effects of cannabidiol. J. Clin. Pharmacol..

[61] Cunha J.M., Carlini E.A., Pereira A.E., Ramos O.L., Pimentel C., Gagliardi R., Sanvito W.L., Lander N., Mechoulam R. (1980). Chronic administration of cannabidiol to healthy volunteers and epileptic patients. Pharmacology.

[62] Crippa J.A., Derenusson G.N., Ferrari T.B., Wichert-Ana L., Duran F.L., Martin-Santos R., Simões M.V., Bhattacharyya S., Fusar-Poli P., Atakan Z. (2011). Neural basis of anxiolytic effects of cannabidiol (CBD) in generalized social anxiety disorder: A preliminary report. J. Psychopharmacol..

[63] Bergamaschi M.M., Queiroz R.H., Chagas M.H., de Oliveira D.C., de Martinis B.S., Kapczinski F., Quevedo J., Roesler R., Schröder N., Nardi A.E (2011). Cannabidiol reduces the anxiety induced by simulated public speaking in treatment-naive social phobia patients. Neuropsychopharmacology.

[64] Leweke F.M., Koethe D., Pahlisch F., Schreiber D., Gerth C.W., Nolden B.M., Klosterkötter J., Hellmich M., Piomelli D. (2009). S39-02 Antipsychotic effects of cannabidiol. Eur. Psychiatry.

[65] Zuardi A.W., Hallak J.E., Dursun S.M., Morais S.L., Sanches R.F., Musty R.E., Crippa J.A. (2006). Cannabidiol monotherapy for treatment-resistant schizophrenia. J. Psychopharmacol..

[66] Zuardi A., Crippa J., Dursun S., Morais S., Vilela J., Sanches R., Hallak J. (2010). Cannabidiol was ineffective for manic episode of bipolar affective disorder. J. Psychopharmacol..

[67] Hallak J.E., Machado-de-Sousa J.P., Crippa J.A., Sanches R.F., Trzesniak C., Chaves C., Bernardo S.A., Regalo S.C., Zuardi A.W. (2010). Performance of schizophrenic patients in the Stroop Color Word Test and electrodermal responsiveness after acute administration of cannabidiol (CBD). Rev. Bras. Psiquiatr..

[68] Killestein J., Hoogervorst E.L., Reif M., Kalkers N.F., van Loenen A.C., Staats P.G., Gorter R.W., Uitdehaag B.M., Polman C.H. (2002). Safety, tolerability, and efficacy of orally administered cannabinoids in MS. Neurology.

[69] Killestein J., Hoogervorst E.L., Reif M., Blauw B., Smits M., Uitdehaag B.M., Nagelkerken L., Polman C.H. (2003). Immunomodulatory effects of orally administered cannabinoids in multiple sclerosis. J. Neuroimmunol..

[70] Zajicek J., Fox P., Sanders H., Wright D., Vickery J., Nunn A., Thompson A, UK MS Research Group (2003). Cannabinoids for treatment of spasticity and other symptoms related to multiple sclerosis (CAMS study): Multicentre randomised placebo-controlled trial. Lancet.

[71] Freeman R.M., Adekanmi O., Waterfield M.R., Waterfield A.E., Wright D., Zajicek J. (2006). The effect of cannabis on urge incontinence in patients with multiple sclerosis: A multicentre, randomised placebo-controlled trial (CAMS-LUTS). Int. Urogynecol. J. Pelvic Floor Dysfunct..

[72] Strasser F., Luftner D., Possinger K., Ernst G., Ruhstaller T., Meissner W., Ko Y.D., Schnelle M., Reif M., Cerny T., Cannabis-In-Cachexia-Study-Group (2006). Comparison of orally administered cannabis extract and delta-9-tetrahydrocannabinol in treating patients with cancer-related anorexia-cachexia syndrome: A multicenter, phase III, randomized, double-blind, placebo-controlled clinical trial from the Cannabis-In-Cachexia-Study-Group. J. Clin. Oncol..

[73] Johnson J.R., Burnell-Nugent M., Lossignol D., Ganae-Motan E.D., Potts R., Fallon M.T. (2010). Multicenter, double-blind, randomized, placebo-controlled, parallel-group study of the efficacy, safety, and tolerability of THC:CBD extract and THC extract in patients with intractable cancer-related pain. J. Pain Symptom Manage..

[74] Brady C.M., DasGupta R., Dalton C., Wiseman O.J., Berkley K.J., Fowler C.J. (2004). An open-label pilot study of cannabis-based extracts for bladder dysfunction in advanced multiple sclerosis. Mult. Scler..

[75] Wade D.T., Robson P., House H., Makela P., Aram J. (2003). A preliminary controlled study to determine whether whole-plant cannabis extracts can improve intractable neurogenic symptoms. Clin. Rehabil..

[76] Notcutt W., Price M., Miller R., Newport S., Phillips C., Simmons S., Sansom C. (2004). Initial experiences with medicinal extracts of cannabis for chronic pain: Results from 34 'N of 1' studies. Anaesthesia.

[77] Berman J.S., Symonds C., Birch R. (2004). Efficacy of two cannabis based medicinal extracts for relief of central neuropathic pain from brachial plexus avulsion: Results of a randomised controlled trial. Pain.

[78] Gomes F.V., Resstel L.B., Guimarães F.S. (2011). The anxiolytic-like effects of cannabidiol injected into the bed nucleus of the stria terminalis are mediated by 5-HT1A receptors. Psychopharmacology (Berl.).

[79] Gomes F.V., Reis D.G., Alves F.H., Corrêa F.M., Guimarães F.S., Resstel L.B. (2012). Cannabidiol injected into the bed nucleus of the stria terminalis reduces the expression of contextual fear conditioning via 5-HT1A receptors. J. Psychopharmacol..

[80] Soares Vde P., Campos A.C., Bortoli V.C., Zangrossi H., Guimarães F.S., Zuardi A.W. (2010). Intra-dorsal periaqueductal gray administration of cannabidiol blocks panic-like response by activating 5-HT1A receptors. Behav. Brain Res..

[81] Resstel L.B., Tavares R.F., Lisboa S.F., Joca S.R., Corrêa F.M., Guimarães F.S. (2009). 5-HT1A receptors are involved in the cannabidiol-induced attenuation of behavioural and cardiovascular responses to acute restraint stress in rats. Br. J. Pharmacol..

[82] Campos A.C., Guimarães F.S. (2009). Evidence for a potential role for TRPV1 receptors in the dorsolateral periaqueductal gray in the attenuation of the anxiolytic effects of cannabinoids. Prog. Neuropsychopharmacol. Biol. Psychiatry.

[83] Biala G., Kruk M., Budzynska B. (2009). Effects of the cannabinoid receptor ligands on anxiety-related effects of d-amphetamine and nicotine in the mouse elevated plus maze test. J. Physiol. Pharmacol..

[84] Bitencourt R.M., Pamplona F.A., Takahashi R.N. (2008). Facilitation of contextual fear memory extinction and anti-anxiogenic effects of AM404 and cannabidiol in conditioned rats. Eur. Neuropsychopharmacol..

[85] Piomelli D., Tarzia G., Duranti A., Tontini A., Mor M., Compton T.R., Dasse O., Monaghan E.P., Parrott J.A., Putman D. (2006). Pharmacological profile of the selective FAAH inhibitor KDS-4103 (URB597). CNS Drug Rev..

[86] Scherma M., Medalie J., Fratta W., Vadivel S.K., Makriyannis A., Piomelli D., Mikics E., Haller J., Yasar S., Tanda G. (2008). The endogenous cannabinoid anandamide has effects on motivation and anxiety that are revealed by fatty acid amide hydrolase (FAAH) inhibition. Neuropharmacology.

[87] Kinsey S.G., O'Neal S.T., Long J.Z., Cravatt B.F., Lichtman A.H. (2011). Inhibition of endocannabinoid catabolic enzymes elicits anxiolytic-like effects in the marble burying assay. Pharmacol. Biochem. Behav..

[88] Welburn P.J., Starmer G.A., Chesher G.B., Jackson D.M. (1976). Effect of cannabinoids on the abdominal constriction response in mice: Within cannabinoid interactions. Psychopharmacologia.

[89] Chesher G.B., Dahl C.J., Everingham M., Jackson D.M., Marchant-Williams H., Starmer G.A. (1973). The effect of cannabinoids on intestinal motility and their antinociceptive effect in mice. Br. J. Pharmacol..

[90] Malfait A.M., Gallily R., Sumariwalla P.F., Malik A.S., Andreakos E., Mechoulam R., Feldmann M. (2000). The nonpsychoactive cannabis constituent cannabidiol is an oral anti-arthritic therapeutic in murine collagen-induced arthritis. Proc. Natl. Acad. Sci. USA.

[91] Sumariwalla P.F., Gallily R., Tchilibon S., Fride E., Mechoulam R., Feldmann M. (2004). A novel synthetic, non-psychoactive cannabinoid acid (HU-320) with anti-inflammatory properties in murine collagen-induced arthritis. Arthritis Rheum..

[92] Schuelert N., McDougall J.J. (2011). The abnormal cannabidiol analogue O-1602 reduces nociception in a rat model of acute arthritis via the putative cannabinoid receptor GPR55. Neurosci. Lett..

[93] Wallace M.J., Wiley J.L., Martin B.R., DeLorenzo R.J. (2001). Assessment of the role of CB1 receptors in cannabinoid anticonvulsant effects. Eur. J. Pharmacol..

[94] Meltzer H.Y., Arvanitis L., Bauer D., Rein W., Meta-Trial Study Group (2004). Placebo-controlled evaluation of four novel compounds for the treatment of schizophrenia and schizoaffective disorder. Am. J. Psychiatry..

[95] Zuardi A.W., Karniol I.G. (1984). Pharmacological interaction between 9-tetrahydrocannabinol and cannabidiol, two active constituents of Cannabis sativa. Ciênc. Cult..

[96] Zuardi A.W., Hallak J.E., Crippa J.A. (2012). Interaction between cannabidiol (CBD) and Δ(9)-tetrahydrocannabinol (THC): Influence of administration interval and dose ratio between the cannabinoids. Psychopharmacology (Berl.).

[97] Klein C., Karanges E., Spiro A., Wong A., Spencer J., Huynh T., Gunasekaran N., Karl T., Long L.E., Huang X.F. (2011). Cannabidiol potentiates Δ^9^-tetrahydrocannabinol (THC) behavioural effects and alters THC pharmacokinetics during acute and chronic treatment in adolescent rats. Psychopharmacology (Berl.).

[98] Guy G.W., Flint M.E. (2004). A single centre, placebo-controlled, four period, crossover, tolerability study assessing, pharmacodynamic effects, pharmacokinetic characteristics and cognitive profiles of a single dose of three formulations of Cannabis Based Medicine Extracts (CBMEs) (GWPD9901), plus a two period tolerability study comparing pharmacodynamic effects and pharmacokinetic characteristics of a single dose of a cannabis based medicine extract given via two administration routes (GWPD9901 EXT). J. Cannabis Ther..

[99] Guy G.W., Robson P. (2003). A Phase I, double blind, three-way crossover study to assess the pharmacokinetic profile of cannabis based medicine extract (CBME) administered sublingually in variant cannabinoid ratios in normal healthy male volunteers (GWPK02125). J. Cannabis Ther..

[100] Kinsey S.G., O’Neal S.T., Long J.Z., Cravatt B.F., Lichtman A.H. (2011). Inhibition of endocannabinoid catabolic enzymes elicits anxiolytic-like effects in the marble burying assay. Pharmacol. Biochem. Behav..

